# Delineation of cell death mechanisms induced by synergistic effects of statins and erlotinib in non-small cell lung cancer cell (NSCLC) lines

**DOI:** 10.1038/s41598-020-57707-2

**Published:** 2020-01-22

**Authors:** Alexander Otahal, Duygu Aydemir, Erwin Tomasich, Christoph Minichsdorfer

**Affiliations:** 1 Anna Spiegel Research Facility, Division of Oncology, Department of Medicine I, Medical University of Vienna, Waehringer Guertel 18-20, 1090 Vienna, Austria; 2 Department of Medical Biochemistry, School of Medicine, Koc University, Istanbul, Turkey; 3 Koc University Research Center for Translational Research (KUTTAM), Istanbul, Turkey

**Keywords:** Non-small-cell lung cancer, Non-small-cell lung cancer

## Abstract

Hydroxymethylglutaryl-coenzyme A (HMG-CoA) reductase inhibitors (statins) have been shown to overcome tyrosine kinase inhibitor (TKI) resistance in epithelial growth factor receptor (EGFR) mutated non-small cell lung cancer (NSCLC) cells *in vivo* and *in vitro*. However, little is known about the putative induction of non-apoptotic cell death pathways by statins. We investigated the effects of pitavastatin and fluvastatin alone or in combination with erlotinib in three NSCLC cell lines and examined the activation of different cell death pathways. We assessed apoptosis via fluorometric caspase assay and poly (ADP-ribose) polymerase 1 (PARP) cleavage. Furthermore, annexinV/propidium iodide (PI) flow cytometry was performed. Small molecule inhibitors benzyloxycarbonyl-Val-Ala-Asp-fluoromethyl ketone (zVAD), necrostatin 1 (Nec1), ferrostatin 1 (Fer1), Ac-Lys-Lys-Norleucinal (Calp1) were used to characterise cell death pathway(s) putatively (co-)activated by pitavastatin/erlotinib co-treatment. Synergism was calculated by additivity and isobolographic analyses. Pitavastatin and fluvastatin induced cell death in EGFR TKI resistant NSCLC cells lines A549, Calu6 and H1993 as shown by caspase 3 activation and PARP cleavage. Co-treatment of cells with pitavastatin and the EGFR TKI erlotinib resulted in synergistically enhanced cytotoxicity compared to pitavastatin monotherapy. Flow cytometry indicated the induction of alternative regulated cell death pathways. However, only co-treatment with mevalonic acid (Mev) or the pan-caspase inhibitor zVAD could restore cell viability. The results show that cytotoxicity mediated by statin/erlotinib co-treatment is synergistic and can overcome erlotinib resistance in K-ras mutated NSCLC and relies only on apoptosis.

## Introduction

Lung cancer is the leading cause of cancer death worldwide and is commonly classified into small and non-small cell lung cancer (SCLC and NSCLC). NSCLC is sub- divided into three major types: (1) squamous cell carcinomas (SCC), (2) adenocarcinomas and (3) large cell carcinomas. In particular, NSCLC accounts for more than 80% of total pulmonary malignancies^1–3^.

EGFR is overexpressed in over 50% of NSCLCs. Oncogenic mutations of the EGFR occur in up to 20% of adenocarcinomas^4^. Targeting EGFR has played a central role in advancing NSCLC research, treatment and patient outcome over the last several years. Osmertinib, afatinib, gefitinib and erlotinib are approved EGFR tyrosine kinase inhibitors (EGFR-TKI) for the treatment of advanced EGFR mutated NSCLC. Erlotinib therapy resulted in a significant improvement in median progression free survival, quality of life, and related symptom control compared to chemotherapy in an EGFR mutated population of advanced and metastatic NSCLC patients (EURTAC trial^5^; OPTIMAL trial^6^; ENSURE trial^7^).

K-Ras mutations lead to primary resistance to EGFR TKIs and are found in 25–30% of adenocarcinomas^8^. In addition to primary resistance, secondary resistance to EGFR TKIs in EGFR-mutant NSCLC patients is commonly acquired through different mechanisms: about 50% of the tumours develop a secondary EGFR mutation in threonine 790 (T790M)^9^, whereas approximately 20% have tumours with amplification of the proto-oncogene MET^10^.

Statins inhibit the rate-limiting step of the mevalonate pathway^11^. Mevalonate is a precursor of several major products including ubiquinone, dolichol, geranylgeranylpyrophosphate and farnesylpyrophosphat^12^. Most statins, such as simvastatin or atorvastatin, are metabolised by CYP3A4, whereas pitavastatin and fluvastatin are oxidised by the CYP2C9 isoenzyme^13–15^. EGFR TKIs like erlotinib or gefitinib are substrates for CYP3A4^16^, which might decrease drug turnover rates resulting in unpredictable or toxic serum levels of statins^17–20^.

Statins are usually well tolerated and have been found to exert multiple pleiotropic effects in micromolar concentrations. Thus, *in vitro* application of statins impacts the mitochondrial respiratory chain, protein glycosylation and post-translational lipid modification of proteins, in particular small G proteins including Ras and Rho^21,22^. Expectedly, statins impair the cell cycle via G1-phase arrest, cell proliferation and differentiation. Furthermore, it was shown that statins induce apoptosis in various tumour cell lines, which has led to a discussion to use statins in anti-cancer treatments^23–27^.

However, in addition to apoptosis, other programmed cell death mechanisms have been described such as necroptosis^28^, ferroptosis^29^ or oncosis^30^. Like the extrinsic pathway of apoptosis, necroptosis is induced via activation of tumour necrosis factor 1 (TNFR1). Active TNFR1 facilitates both, apoptosis via Fas-associated protein with death domain (FADD) and caspase 8 as well as necroptosis via receptor interacting protein kinases 1 and 3 (RIPK1 and RIPK3). Interestingly, caspase 8 inhibits necroptosis via degrading RIPK3^31^. The pan-caspase inhibitor zVAD halts apoptosis, whereas Nec1 inhibits RIPK1 and necroptosis^32^. In contrast to apoptosis, cellular components are not degraded during necroptosis. Necroptotic effector mechanisms include overproduction of reactive oxygen species (ROS) and perforation of the cell membrane, leading to leakage of intracellular molecules into the extracellular space, ultimately promoting inflammation and immune responses^33^. Ferroptosis is distinct from other regulated cell death pathways as it can neither be prevented by zVAD nor Nec 1^34^. Experimental and clinical drugs can interfere with iron metabolism and induce lipid peroxidation, which can be inhibited via Fer1^35,36^. Another caspase-independent cell death pathway is called oncosis, which is characterised by cell swelling and loss of membrane integrity indicated by permeability for propidium iodide^30^. It relies on activation of calpain^37^, which can be inhibited by Calp1^38^.

Up to now it is not known if statins activate different types of cell death mechanisms, other than apoptosis, or if a combination treatment of erlotinib and statins could exploit activation of additional cell death pathways and thereby lead to a more pronounced cytotoxic effect on tumour cells.

Atorvastatin and simvastatin have been shown to increase the cytotoxic effect of EGFR TKIs in mouse models^39,40^. However, they share similar metabolic pathways with erlotinib, which may lead to toxic serum levels of statins resulting in rhabdomyolysis^20^. Therefore, the primary aim of this study was to investigate the cytotoxic effects of pitavastatin and fluvastatin, which are metabolised by a different subset of CYP enzymes, alone and in combination with erlotinib, using three different human NSCLC cell lines. Additionally, we investigated if potential synergistic effects of the combined treatment may rely on the concurrent activation of cell death pathways other than apoptosis.

## Methods

### Cell culture

Experiments were carried out with human lung adenocarcinoma cell lines A549 (ATCC CCL_185), Calu6 (ATCC HTB-56) and NCI-H1993 (ATCC CRL-5909). All cell lines were obtained from American Type Cell Culture Collection (ATCC) and cultured in DMEM growth medium (GIBCO #31966-21), supplemented with 10% heat-inactivated foetal bovine serum (FBS) and 1% antibiotics (penicillin, streptomycin). Cells were kept at 37 °C and 5% CO_2_ in the incubator and were passaged at 80–90% confluence every 2–3 days to maintain continuous logarithmic growth. All cell lines studied in this work were erlotinib resistant and EGFR wild type (Table 1). Cells were treated with pitavastatin calcium (SelleckChem, #S1759), fluvastatin sodium (SelleckChem, #S1909) and erlotinib hydrochloride (SelleckChem, #S1023).

**Table 1:** Human NSCLC cell lines harbouring different genetic mutations examined in the study.

Cell line	EGFR	K-Ras	Met	p53	Erlotinib
A549	Wildtype	Mutated	Wildtype	Wildtype	Resistant
Calu6	Wildtype	Mutated	Wildtype	Mutated	Resistant
H1993	Wildtype	Wildtype	Amplified	Mutated	Resistant

### Preparation of lysates from human NSCLC cell lines

Cells were grown in 25 cm^2^, polystyrene, canted neck cell culture flasks until 70% of confluence was reached and afterwards incubated with indicated drugs for up to 72 hours (h) at 37 °C. Afterwards, the flasks were washed in ice-cold phosphate buffered saline (PBS) 3 times on ice. Cells were lysed with 300 µl caspase lysis buffer (25 mM HEPES, 5 mM EDTA, 1 mM EGTA, 5 mM MgCl_2_, 1% leupeptin, pefablock and aprotinin), followed by 15 minutes incubation on ice, scraping off and transferring into 1.5 ml centrifuge tubes. Samples were sonicated for 10 seconds and then centrifuged at full speed for 25 minutes at 4 °C. Samples were stored at −80 °C until analysis.

### Protein determination

After harvesting cells in caspase lysis buffer or Thermo Scientific IP Lysis Buffer (#87787) containing Thermo Scientific Pierce Phosphatase Inhibitor Tablets (#88667), soluble protein concentrations in lysates were quantified by Bradford assay using bovine serum albumin as standard. Caspase lysis buffer (25 mM HEPES, 5 mM EDTA, 1 mM EGTA, 5 mM MgCl_2_) was used as a background control. After adding 250 µl Thermo Scientific Pierce Coomassie substrate per well, samples were incubated for 5 minutes at room temperature and absorbance was measured at 450 nm on a Thermo Scientific Multiskan Spectrum v1.2 with SkanIt Software 2.4.4.

### Caspase-3 activity assay

Cells were exposed to increased concentrations of fluvastatin (0.1–100 µM), pitavastatin (0.1–100 µM) and erlotinib (0.05–10 µM) alone and each statin in combination with erlotinib for up to 72 h at 37 °C and 5% CO_2_. Cells were harvested as described above and 30–80 µl of lysate was incubated with the same amount of caspase reaction buffer (40 mM HEPES, pH 7.5, 20% glycerol, 4 mM DTT) containing 50 µM of the 7-amino-4-trifluoro-methylcoumarin (AFC)-conjugated substrate (ENZO, #ALX-260–032-M005). Samples were incubated for 90 minutes at 37 °C in the dark, then fluorescence was measured at 535 nm using a fluorescence plate reader (BertholdTech Tristar). Lysis buffer containing equivalent amounts of caspase reaction buffer was used as a background control.

### Western Blot

After protein quantification as described above, 30–70 µg protein from each sample was diluted with 5x Laemmli buffer (10% SDS, 50% glycerol, 0.3 M Tris-Hcl (pH 6.8), 0.05% bromphenolblue), containing 10% of β-mercaptoethanol, and then kept for 2 minutes at 95 °C. Samples were separated on 10% SDS-PAGE with 5% stacking gel at 90 V. Proteins were wet transferred to PVDF membrane for 1 hour at 150 mA at 4 °C. Membranes were blocked with 5% BSA in phosphate buffered saline/0,1% Tween (PBS-T) or Tris buffered saline/0,1% tween (TBS-T) for 1 hour and probed with primary antibodies (anti-phospho-Akt Ser473 #4060, anti-Akt #4691, anti-phospho-ERK1/2 (Thr202/Tyr204) #4370, anti-ERK1/2 #4695, anti-PARP #9542, anti-β-actin D6A8 #8457, anti-caspase 1 #2225 T or anti-tubulin #T4026; all purchased from Cell Signalling, except anti-tubulin which was purchased from Sigma-Aldrich) which were diluted in 5% BSA (1:1000) at 4 °C overnight. Membranes were washed 3 times with PBS-T or TBS-T and incubated with horseradish peroxidase-coupled secondary antibody (Cell Signalling #7074) which was diluted in 5% BSA (1:10,000) for 1 hour at room temperature. Next, membranes were washed three times for 5 min with PBS-T or TBS-T and incubated with enhanced chemiluminescent reagent for 2 minutes, followed by developing membranes in Chemidoc XRS+ with Image Lab Software up to 20 minutes. Band densitometry was performed as described previously^41^.

### Flow cytometry

Cells were split into 25 cm^2^ flasks as described above and grown until 70% confluence. Cells were treated with increased concentrations of pitavastatin (1–100 µM) alone and in combination with erlotinib (1–5 µM) for up to 72 h at 37°C and 5% CO_2_. Afterwards, cell supernatants were transferred to 5 ml polystyrene tubes. Cells were washed twice with PBS, detached using trypsin/EDTA and combined with the appropriate supernatant, followed by centrifugation at 4 °C for 5 min at 300 × g. ENZO Annexin V - fluoresceinisothocyanate (FITC) apoptosis detection kit (#ALX-850-020-KI01) was used for labelling cells, according to the manufacturer’s recommendations. Fluorescent cells were measured using a BD LSRFortessa and percentages of labelled cells were determined by FlowJo vX.0.7.

### Inhibitor screen

Growth media of cells treated with erlotinib (5 µM) and pitavastatin (10 µM) alone or in combination were supplemented with inhibitors targeting apoptosis (zVAD, APExBIO #A1902-10, 100 µM), necroptosis (Necrostatin 1, Nec, Merck #504297, 10 µM), ferroptosis (Ferrostatin 1, Fer1, APExBIO #A4371-25, 10 µM) or calpain protease (Ac-Lys-Lys-Norleucinal, Calp1, Sigma-Aldrich #A6185, 10 µM). Mevalonic acid was used as rescue control. Cells (1.5 * 10^5^) were seeded in 12 well plates 24 h prior to adding the supplements. After 72 h, cells were collected for flow cytometry as described.

### Fluorometric calpain activity assay

To confirm the inhibitory effect of Calp1 we used a commercially available Calpain Activity Fluorometric Assay Kit (Sigma-Aldrich, #MAK228). The assay reaction was prepared by following the manufacturer’s instruction except for adding CALP1 either alone or to the Active Calpain (Sigma-Aldrich, #MAK228D) in a final concentration of 10 µM. After one hour of incubation at 37 °C, fluorescence was measured on a multiwell plate reader (Varioskan LUX, Thermo Fisher). The experiment was repeated three times independently.

### 3-(4,5-Dimethylthiazol-2-yl)-2,5-diphenyltetrazoliumbromid (MTT) assay

Five-thousand cells per well were seeded in a 96-well plate and left to adhere overnight. After cell adherence, the medium was exchanged with medium supplemented with 10 µM erastin (SelleckChem, #S7242), 10 µM Fer 1 or a combination of them. Cells receiving the inhibtor were pre-treated for 1 hour. After 48 h, CellTiter Blue reagent (Promega, #G8080) was added and incubated for 2–4 h at 37 °C. Fluorescence was recorded on on a multiwell plate reader (Varioskan LUX, Thermo Fisher). The experiment was repeated three times independently.

### Analysis of drug interaction

We employed three approaches to assess the extent of synergy between pitavastatin (Pita) and erlotinib (Erlo) treatment. First, cytotoxicity was evaluated by determining the CDI (coefficient of drug interaction) via the formula CDI = S(Erlo + Pita)/[S(A) * S(B)] relating survival rates (S) in presence of both drugs versus the product of either drug alone. Antagonism, additivity or synergy is represented by values >1, = 1 or <1, respectively. A CDI below 0.7 indicates significant synergism^42,43^. Secondly, Bliss independence was calculated^44,45^. The effect rates E(Erlo) and E(Pita), expressed as percent of dead cells at matching concentrations between 0–100 µM, were normalised to 100% cell death to yield the fractional response. Then, baseline cell death unrelated to treatment was subtracted as observed in untreated samples, before effect rates were normalised to maximum response per cell line and treatment. In case of additive effects, the effect rate of the combination of erlotinib and pitavastatin (E_exp_) is expected to equal the sum of individual effect rates minus their product as given by the formula E_exp_ = E(Erlo) + E(Pita) − E(Erlo) * E(Pita). Using nonlinear regression, employing the user-defined model given in^46^, the expected effect rates were fitted and compared to observed effect rates [E_obs_ = E(Erlo + Pita)] of the combination treatment.

Thirdly, linear isobolographic analysis was performed by plotting the half maximal effective concentration (EC50) of single drug treatments as intercepts of an isobole with the axes of an *xy* plot^47^. The observed EC50 of pitavastatin in presence of 5 µM erlotinib was then plotted on the graph. If the effect rate of the combination treatment lies on, above or below the isobole, the drug combination is additive, antagonistic or synergistic, respectively^48,49^.

### Dose response analysis

Percentages of dead cells as obtained via flowcytometry were plotted against tested drug combinations and fitted non-linearly using the log(agonist)-response model with variable slope via GraphPad Prism version 5. Bottom and top constraints were used and set to greater than zero and less than 100, respectively. EC50 values of the single agonists or the agonist combination were derived from the fitted curve.

### Statistical analysis

GraphPad Prism version 5 was used for statistical analyses and generating data plots. Data were analysed via either two-tailed unpaired t-test or one-way ANOVA followed by Dunett’s or Tukey’s multiple comparison tests, as noted in the figure legends. Two-sided p-values below α = 0.05 were considered significant.

## Results

### Statins eliminate NSCLC cells via apoptosis mediated by dose-dependent inhibition of the mevalonate pathway

**Figure 1:**
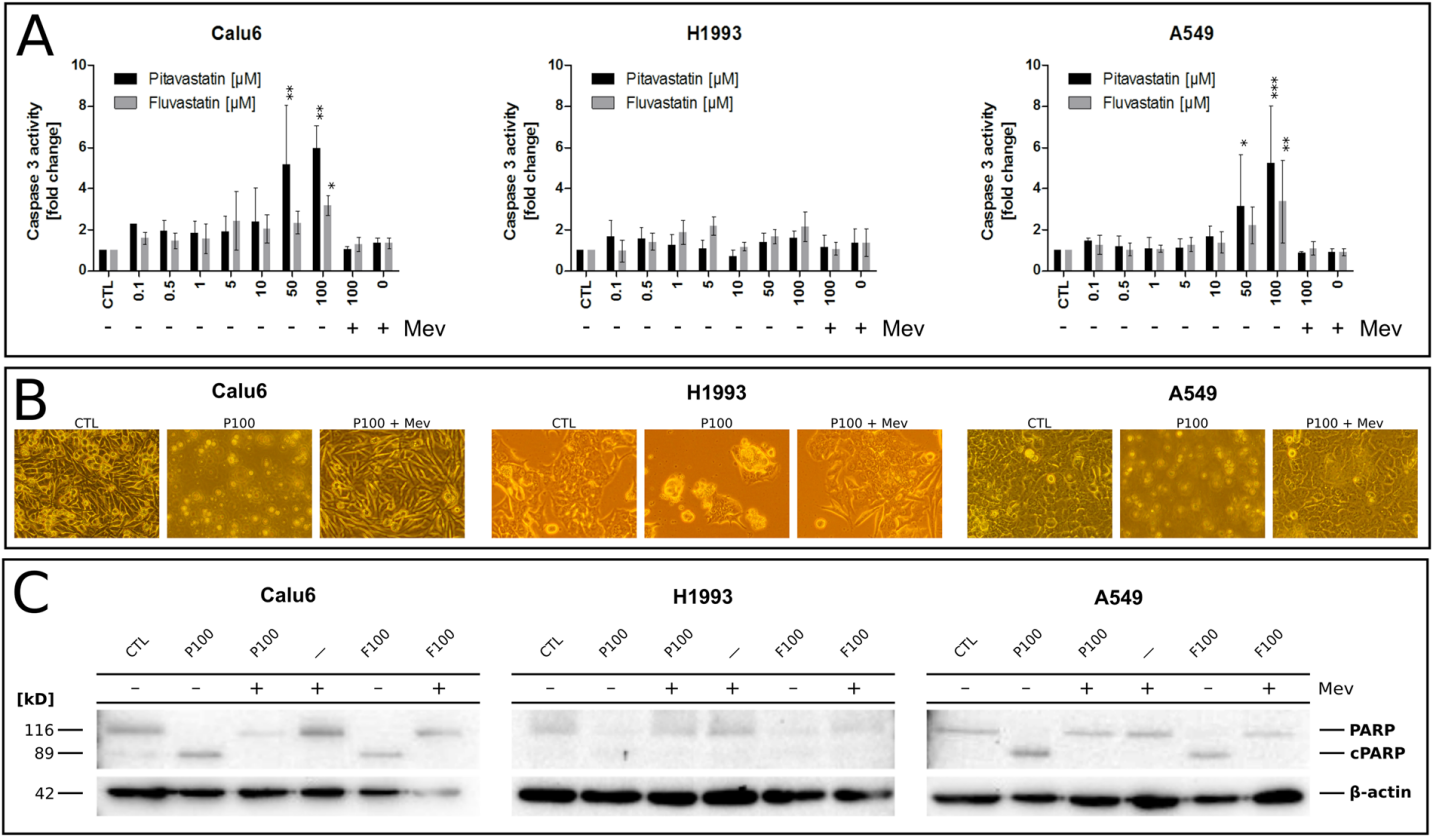
Statin-induced apoptosis and its lipid dependency in NSCLC cells. (**A**) Evaluation of caspase 3 activation in cells treated with up to 100 µM of either pitavastatin or fluvastatin for 48 h. Mevalonic acid (Mev, 1 mM) was used as rescue control counteracting statin-mediated intracellular depletion of sterol precursors. Mev and statin co-treated cells showed caspase 3 activity indistinguishable from untreated cells. One-way ANOVA, α = 0.05; Calu6 (pitavastatin): F(9,22) = 4.805, p = 0.0013; Calu6 (fluvastatin): F(9,16) = 2.207, p = 0.0801; H1993 (pitavastatin): F(9,26) = 1.337, p = 0.2664; H1993 (fluvastatin): F(9,21) = 2.794, p = 0.0252; A549 (pitavastatin): F(9,30) = 4.152, p = 0.0015; A549 (fluvastatin): F(9,18) = 3.411, p = 0.0128. Data are given as arithmetic mean of fold change relative to untreated control (CTL) ± SD from three independent experiments. Asterisks denote statistical significance as determined via Dunnett’s multiple comparison test compared to untreated control (CTL): *p < 0.05; **p < 0.01; ***p < 0.001. (**B**) Morphological changes associated with statin exposure compared to untreated cells or mevalonate rescue control. (**C**) Assessment of PARP cleavage (cPARP) in cells treated with 100 µM pitavastatin (P) or fluvastatin (F) alone or in combination with 1 mM mevalonic acid (Mev) for 48 h via 10% SDS-PAGE and Western Blot. β-actin was used as loading control.

Treatment of lung cancer cell lines with pitavastatin or fluvastatin at concentrations between 0.1–100 µM for 72 h led to caspase 3 activation and PARP cleavage as well as typical morphological changes like rounding of the cells and detachment from the surface, indicating apoptosis. Both statins activated caspase 3 significantly at 50 or 100 µM in Calu6 or A549 cells, but failed to do so in H1993 cells (Fig. 1A–C). However, the co-administration of mevalonic acid (Mev) prevented statin-induced morphological alterations, caspase 3 activation, and the cleavage of PARP. Collectively, these results show that human NSCLC cells harbouring different genetic mutations are susceptible to statin-induced cell death *in vitro*, which is mediated by blocking the mevalonate pathway through inhibition of HMG-CoA reductase.

### Synergistic cytotoxicity of pitavastatin and erlotinib in NSCLC cells *in vitro*

**Figure 2:**
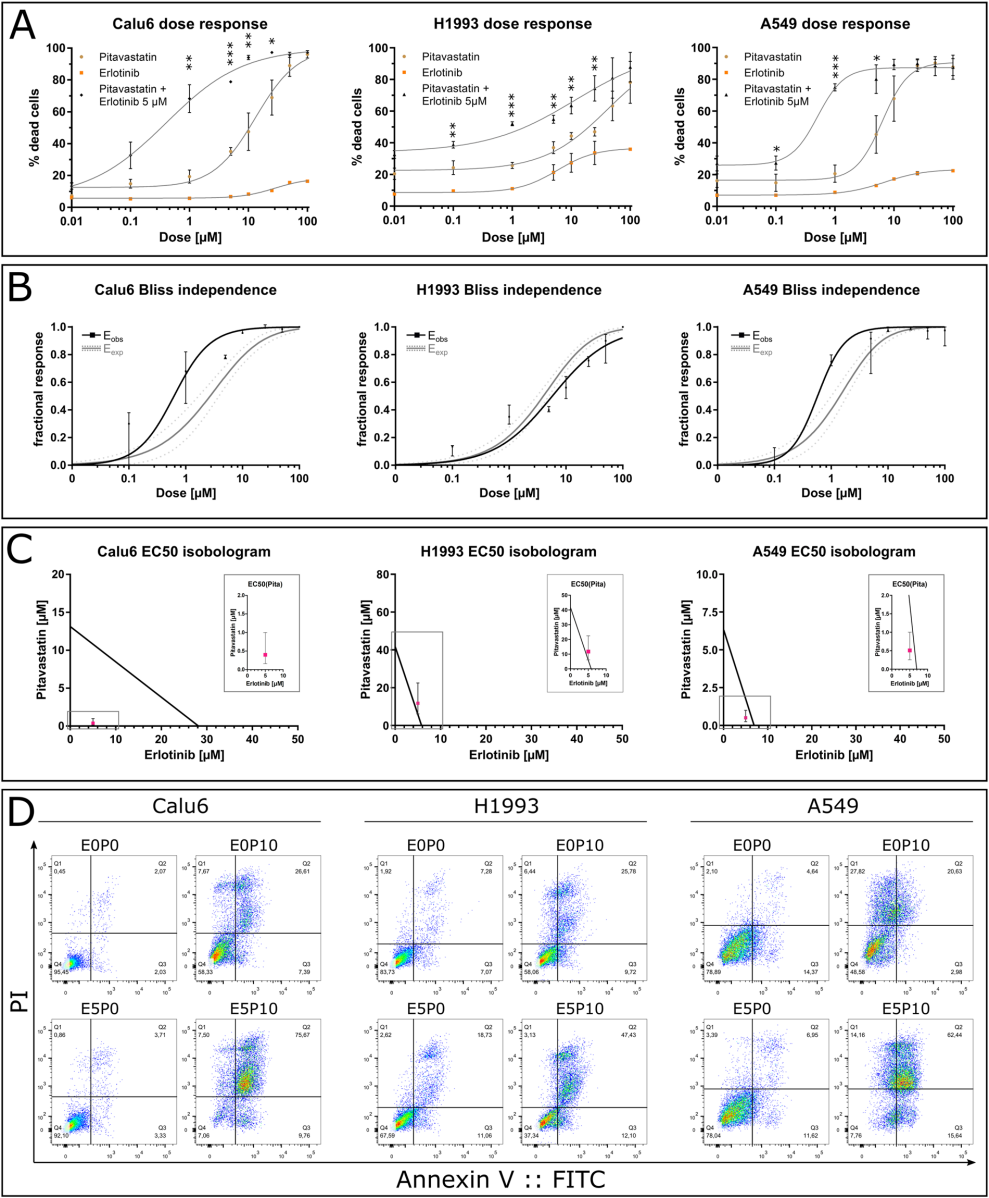
Synergistic induction of cell death as a function of pitavastatin concentration in combination with the EGFR TKI erlotinib in NSCLC cell lines. (**A**) Dose response curves of indicated cell lines treated with increasing concentrations of pitavastatin and/or erlotinib (5 µM). Proportions of dead cells were determined via flow cytometry. EC50 values were calculated using Graph Pad Prism (Table 2). Data are given as arithmetic mean ± SD from three independent experiments. Asterisks denote statistical significance as determined via unpaired two-tailed t-test comparing pitavastatin-treated to pitavastatin and erlotinib co-treated cells at matching concentrations: *p < 0.05; **p < 0.01; ***p < 0.001. (**B**) Bliss independence analysis of drug synergy. Expected fractional effect rate calculated from single drug treatments (grey curve with 95% confidence band) are compared with observed fractional effect rates (black squares and curve). The observed cytotoxicity (black curve) was higher than expected (grey curve) indicating a synergistic rather than additive cytotoxicity of pitavastatin/erlotinib co-treatment in Calu6 and A549 cells compared to H1993 cells. Data are derived from panel A and shown as median and 95% CI. (**C**) EC50 isobolographic analysis showing the decrease of EC50 of pitavastatin in presence of 5 µM erlotinib [EC50(Pita)]. Data are median and 95% CI. The isobole was generated using data from Table 2. If the effect rate of the combination treatment lies on, above or below the isobole, the drug combination is additive, antagonistic or synergistic, respectively. (**D**) Representative dot plots of annexin V (AV)/PI labelled cells treated with the indicated drug combinations: untreated (E0P0), pitavastatin 10 µM (E0P10), erlotinib 5 µM (E5P0) and erlotinib 5 µM/pitavastatin 10 µM co-treatment (E5P10). Prominent populations of AV/PI double positive cells appear in presence of pitavastatin (E0P10). This is even more pronounced in combination with erlotinib (E5P10), but absent in untreated (E0P0) or erlotinib-only exposed cells (E5P0). Dot plots were generated using FlowJo vX.0.7.

Combination treatment was performed with erlotinib and pitavastatin. As expected, erlotinib alone was ineffective at inducing cell death in the erlotinib resistant cell lines Calu6, H1993 and A549, as determined via flow cytometry (Fig. 2A). Statin-induced cytotoxicity was significantly enhanced by 5 µM erlotinib, lowering the EC50 of pitavastatin in all three cell lines (Fig. 2A, Table 2). Coefficients of drug interaction (CDI) were calculated as described in Materials and Methods (Table 3). CDI evaluation revealed that combining drugs enhanced cytotoxicity when using between 1–50 µM and 1–10 µM pitavastatin and 5 µM erlotinib in Calu6 and A549 cells, respectively. However, the 0.7 cut-off for synergistic interaction was not reached in H1993 cells.

**Table 2:** EC50 values of erlotinib as well as pitavastatin alone or in combination with 5 µM erlotinib determined via flow cytometry.

Cell line	Erlotinib	Pitavastatin	Pitavastatin + Erlotinib (5 µM)	Significance
A549	6.938 µM (5.752–8.368)	6.344 µM (5.026–8.008)	0.509 µM (0.258–1.003)	t(4) = 3.765; p = 0.02
Calu6	28.19 µM (20.08–39.59)	13.13 µM (8.09–21.31)	0.398 µM (0.1587–0.9968)	t(4) = 4.318; p = 0.0125
H1993	5.807 µM (4.372–9.712)	41.77 µM (2.811–620.8)	11.69 µM (0.951–143.8)	t(4) = 11.75; p = 0.0003

Values are given as median with CI 95% determined via fitting a variable slope dose response curve to data of three experiments using GraphPad as described in the Methods section. Statistical significance of the dose response (EC50) shift of pitavastatin alone and in combination with erlotinib was determined via unpaired two-tailed t-Test.

**Table 3:** Coefficients of drug interaction (CDI) calculated from data shown in Fig. 2A. Anatagonism, additivity or synergy is represented by values larger than 1, equal to 1 or below 1.

Pitavastatin [µM]	Calu6	H1993	A549
0	1,00 ± 0,05	0,92 ± 0,05	0,94 ± 0,11
0.1	0,83 ± 0,17	0,90 ± 0,04	0,92 ± 0,01
1	0,42 ± 0,20	0,73 ± 0,03	0,35 ± 0,01
5	0,35 ± 0,03	0,92 ± 0,16	0,41 ± 0,14
10	0,12 ± 0,02	0,91 ± 0,20	0,48 ± 0,28
25	0,10 ± 0,03	0,74 ± 0,29	1,21 ± 0,57
50	0,47 ± 0,07	N.D.	N.D.
100	0,94 ± 0,23	0,94 ± 0,53	1,30 ± 0,77

A CDI below 0.7 indicates significant synergy. Data are given as mean ± SD of three experiments. N.D. not determined.

According to bliss independence analysis, co-treatment with 5 µM erlotinib resulted in synergistic cytotoxicity with pitavastatin at doses between 1–25 µM and 1–5 µM for Calu6 and A549 cells, respectively. For H1993 only limited synergy was determined (Fig. 2B).

In parallel, an isobolographic analysis was performed, which proved the synergistic effects in A549 and Calu6 cells (Fig. 2C). The EC50 of pitavastatin in the presence of 5 µM erlotinib (pink squares) is located below the isobole for Calu6 and A549. In contrast, the cytotoxic effect in H1993 cells is only additive, as the EC50 95% CI range coincides with the isobole. These results support the hypothesis that the cytotoxicity of pitavastatin/erlotinib co-treatment is of synergistic nature in A549 and Calu6 cells.

### Cytotoxicity of pitavastatin-erlotinib co-treatment relies only on apoptosis

**Figure 3:**
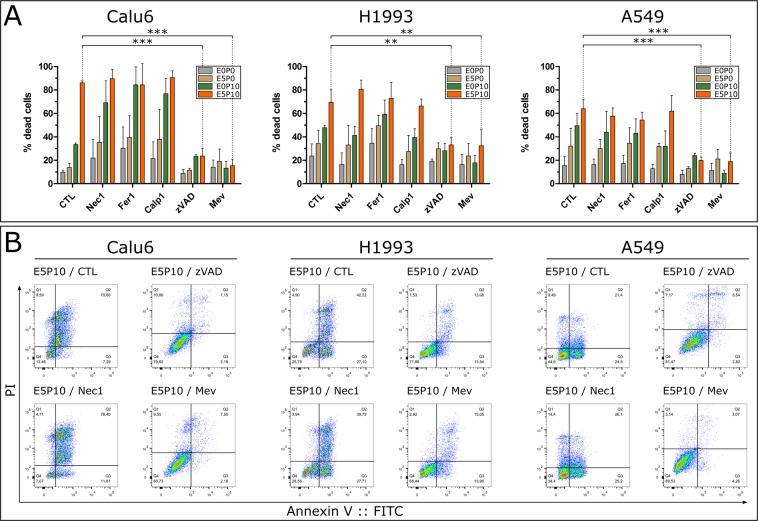
Cell death inhibitor screen. (**A**) NSCLC cells were treated with combinations of erlotinib and pitavastatin: untreated (E0P0), pitavastatin 10 µM (E0P10), erlotinib 5 µM (E5P0) and erlotinib 5 µM/pitavastatin 10 µM co-treatment (E5P10). Cells were co-treated with either 10 µM Nec 1, 10 µM Fer1, 10 µM Calp1, 100 µM zVAD or 1 mM Mev. Proportions of dead cells were determined via flow cytometry. Cell death of E5P10 treated cells was significantly inhibited by zVAD or Mev in Calu6 (F(5,11) = 36.71, p < 0.0001; q(zVAD) = 8.153, q(Mev) = 8.255), H1993 (F(5,11) = 13.81, p = 0.0001; q(zVAD) = 4.707, q(Mev) = 4.553) and A549 (F(5,11) = 20.37, p < 0.0001; q(zVAD) = 6.714, q(Mev) = 6.859) cells. Statistical significance was determined via one-way ANOVA and Dunnett’s test. Data are given as arithmetic mean ± SD from three independent experiments. *p < 0.05; **p < 0.01; ***p < 0.001. (**B**) Representative flow cytometry dot plots of E5P10 treated cells unsupplemented (CTL) or supplemented with the indicated inhibitors (Mev, Nec1, zVAD) from different experiments. Dot plots were generated using FlowJo vX.0.7.

The combination of pitavastatin and erlotinib resulted in the appearance of large populations of annexinV::FITC/PI-positive cells (Fig. 2D). PI requires a leaky plasma membrane to enter a cell, which is usually not the case in purely apoptotic cells, but may be indicative of other forms of cell death^30,50^. These observations led to the hypothesis that alternative cell death pathways might be responsible for the synergistic drug interaction. An inhibitor screen was performed to investigate the activation of other possible cell death mechanisms. However, cell death was effectively inhibited only by zVAD and mevalonic acid in all evaluated cell lines. In contrast, no significant reduction in dead cells was observed in response to Nec1, Fer1 and Calp1 in any cell line (Fig. 3A). In addition, supplementing media of E5P10 treated cells with Mev restored cell viability (Fig. 3B). In conclusion, the enhanced cell death induced by pitavastatin/erlotinib co-treatment is driven by apoptosis rather than necroptosis, ferroptosis or oncosis and relies on the inhibition of the mevalonate pathway. To prove that the applied inhibitors Fer1, Calp1 and Nec 1 were functional, they were tested via inhibition of ferroptosis induced by erastin in A549 cells, a fluorometric calpain activity assay *in vitro*, and inhibition of necroptosis triggered by TNFα and cylcoheximide in Jurkat cells (as described earlier^51^), respectively (Fig. S1).

### Inhibitory effects of combination therapy are mediated by ERK/MAPK and PI3K/AKT signalling

**Figure 4:**
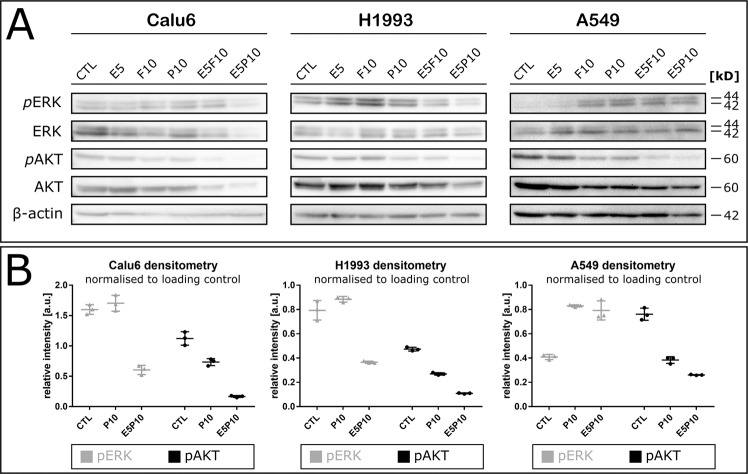
Co-treatment of NSCLC cell lines affects ERK1/2 and AKT signalling. (**A**) Cells were treated with 5 µM erlotinib (E), 10 µM pitavastatin (P) or fluvastatin (F) alone or in combination with 5 µM erlotinib for 72 h. β-actin was used as loading control, one of two replicate blots is shown. (**B**) Quantification of band intensities from (**A**) by densitometric analysis via ImageJ using the GelAnalyzer plugin as described^41^. Data represent arithmetic mean of three technical quantification replicates ± SD. F, fluvastatin; P, pitavastatin.

To highlight the possible mechanisms underlying apoptotic effects of combining statins with erlotinib, we investigated the activation of ERK/MAPK and PI3K/AKT signalling pathways in response to combination therapy in Calu6, H1993 and A549 cells via Western Blot (Fig. 4A).

Total ERK remained constant in the presence of either statin, whereas the change of phospho-ERK1/2 levels (pERK) differed between cell lines. Phosphorylation was marginally decreased upon statin treatment in Calu6 and H1993 cells but increased in A549. Total AKT was constant, irrespective of treatment. The phosphorylation of AKT (pAKT) at Ser473 remained unchanged by erlotinib alone, but was slightly reduced upon statin treatment. However, co-treatment of cells with pitavastatin and erlotinib resulted in a strong reduction of pAKT; below 50% compared to untreated cells (CTL) in all cell lines as determined via band densitometry (Fig. 4B).

## Discussion

Despite recent advances in NSCLC therapy, the prognosis of stage IV disease is still poor. Therefore, various studies are focusing on developing new therapeutic approaches and enhancing the efficacy of available drugs for the treatment of lung cancer^52,53^. Statins exhibit anti-tumour activity via growth inhibition, activation of apoptosis and inhibition of cell motility^19,23^. The potential therapeutic role of simvastatin, lovastatin or atorvastatin was demonstrated before and shown to stimulate both apoptosis and growth inhibition in various human NSCLC cells in a dose- and time-dependent manner^12,17,18,39,40^. In our study, we confirmed the *in vitro* cytotoxic efficacy of fluvastatin and pitavastatin in combination with erlotinib on EGFR TKI resistant human lung adenocarcinoma cell lines A549, Calu6 and H1993. It has previously been shown that the cytotoxic effects of statins are dependent on the mevalonate pathway^23,25,27,54^. This is consistent with our data as co-administration of 1 mM mevalonic acid abrogated pitavastatin or fluvastatin induced activation of caspase-3, changes in cell morphology and PARP cleavage (Fig. 1A–C).

Recent *in vivo* studies in mice showed that lovastatin can overcome gefitinib resistance in NSCLC cells harbouring a K-Ras mutation via inhibition of the MAPK and PI3K/AKT pathways^55^. Similarly, atorvastatin and simvastatin can overcome gefitinib resistance in K-Ras or T790M mutated NSCLC cells through impairing AKT and ERK activity^39,40^. Furthermore, another study reported increased survival of patients with K-Ras driven NSCLC when using combination treatment with erlotinib or gefitinib and atorvastatin or simvastatin^56^. These results are in line with our findings as drug interaction analyses showed that the increased cytotoxicity elicited by pitavastatin/erlotinib combination treatment is synergistic (Fig. 2B,C). We tested our data via calculating coefficients of drug interaction (CDI) (Table 3) to determine the most effective concentration ranges. Bliss independence (Fig. 2B) and isobolographic analysis (Fig. 2C) indicate that the drugs act synergistically while erlotinib alone is ineffective. Furthermore, we showed that the elicited synergy is cell-line specific and the highest activity is reached in K-Ras driven NSCLC cell lines Calu6 and A549. Statins lead to reduced availability of prenylation precursors; consequently K-Ras membrane association is impaired^57^. This causes the loss of constitutive K-Ras signalling. Presence of erlotinib, which binds the wildtype EGFR in Calu6 and A549 cells, suppresses EGFR activation. This might prevent residual EGFR-mediated proliferative signalling. It has to be pointed out that the concentrations used in our *in vitro* experiments for pitavastin (0,1–100 µM) are not achievable with routine statin therapy. Statin therapy for hypercholesterinaemia results in plasma concentrations in the range of 10–100 nM^58,59^. Consequently, the effects we observed *in vitro* will not be elicited by routine statin therapy. Nevertheless, high-dose statin therapy (15–45 mg/kg/d), which results in peak plasma levels of around 4 µM, is feasible and toxicity can be controlled by the supplementation of ubiquinone^60,61^. This is comparable with the dose range we used in our *in vitro* study.

However, the observed cytotoxicity of the combination treatment was less effective in MET driven H1993 cells. Amplification of MET oncogene activates EGFR and/or ERBB3 via receptor crosstalk^10,62,63^. This might dampen the activity of the combination treatment. Replacing erlotinib or supplementing with a MET inhibitor^64^ in a statin combination treatment might yield similar synergistic effects in MET driven NSCLC cells.

Several studies suggest that statin-mediated anti-cancer effects are caused by the inhibition of EGFR downstream signalling pathways in various cancer cells such as lung, prostate, breast, head and neck^65^. The Ras/MAPK and PI3K/AKT cascades are major signalling networks triggered via EGFR-activation^66^. Genetic alterations in regulatory proteins involved in these pathways, such as Ras, are closely related with tumorigenesis especially in epithelial tissue-derived cancers such as lung carcinomas^67^.

Previous studies demonstrated that constitutive activation of the PI3K/AKT signalling cascade is associated with resistance to EGFR TKIs^10^. Thus, inhibition of both pathways concurrently is considered to induce apoptosis and eliminate various types of cancer cells^68,69^.

According to our data, co-treatment with statins and erlotinib inhibited the AKT pathway in Calu6, H1993 and A549 cells (Fig. 4). The synergistic shutoff of the EGFR/K-Ras signalling route hinders activation of AKT via K-Ras^66^. Apparently, loss of AKT signalling is sufficient to induce apoptosis in response to statin and erlotinib exposure.

Various cell death signalling pathways exist besides apoptosis. Up to now, only the activation of apoptosis by statins has been thoroughly investigated^25,27^. To our knowledge, it has never been investigated if statins exploited cell death mechanisms other than apoptosis in tumour cells. In the present study, we could exclude the concurrent activation of other regulated cell death pathways rather than apoptosis as driving force for the synergistic effects (Fig. 3A,B). The co-incubation with a panel of cell death inhibitors failed to rescue cells treated with pitavastatin alone or in combination with erlotinib. Only the treatment with a pan-caspase inhibitor, as well as supplementation with mevalonic acid, inhibited the cytotoxic effects of the combination treatment (Fig. 3A). Taken together, our data indicate that cell death mediated by pitavastatin and erlotinib strictly relies on the mevalonate pathway and the activation of apoptosis.

## Conclusion

We investigated *in vitro* anti-cancer properties of pitavastatin and fluvastatin alone and in combination with erlotinib on three human NSCLC cell lines harbouring different genetic mutations. In summary, we confirmed that both statins activate apoptosis in NSCLC cell lines in vitro. Co-administration of pitavastatin with erlotinib synergistically increased pitavastatin cytotoxicity, especially in K-Ras mutated cell lines. Nevertheless, the effects were weaker in MET-driven EGFR-TKI resistance. Finally, we could exclude the activation of alternative cell death pathways other than apoptosis by pitavastatin alone or in combination with erlotinib.

## Supplementary information

Figure S1.

Western Blots.
