# Supplementary material for: Delineation of cell death mechanisms induced by synergistic effects of statins and erlotinib in non-small cell lung cancer cell (NSCLC) lines
Source: Sci Rep. 2020 Jan 22;10:959. doi: 10.1038/s41598-020-57707-2 (PMC6976657; doi:10.1038/s41598-020-57707-2)
Supplement: Supplementary file 1 — Figure S1. [file 41598_2020_57707_MOESM1_ESM.pdf]

Delineation of cell death mechanisms induced by synergistic effects of statins and erlotinib in non-small cell lung cancer cell (NSCLC) lines

Alexander Otahal[1], Duygu Aydemir[1,2], Erwin Tomasich[1], Christoph Minichsdorfer[1]

[1] Anna Spiegel Research Facility, Internal Medicine I, Oncology Division, Medical University of Vienna, Lazarettgasse 14, 1090 Vienna

[2] Department of Medical Biochemistry, School of Medicine, Koc University, Istanbul, Turkey, Koc University Research Center for Translational Research (KUTTAM), Istanbul, Turkey; before 2016: Anna Spiegel Research Facility, Internal Medicine I, Oncology Division, Medical University of Vienna, Lazarettgasse 14, 1090 Vienna

Contributing author, alexander.otahal@meduniwien.ac.at (ORCID: 0000-0003-3705-0039)

Contributing author, daydemir16@ku.edu.tr (ORCID: 0000-0002-6449-2708)

Contributing author, erwin.tomasich@meduniwien.ac.at

Corresponding author, christoph.minichsdorfer@meduniwien.ac.at

(ORCID: 0000-0002-5455-9139)

Figure S1 – Supplemetary figure

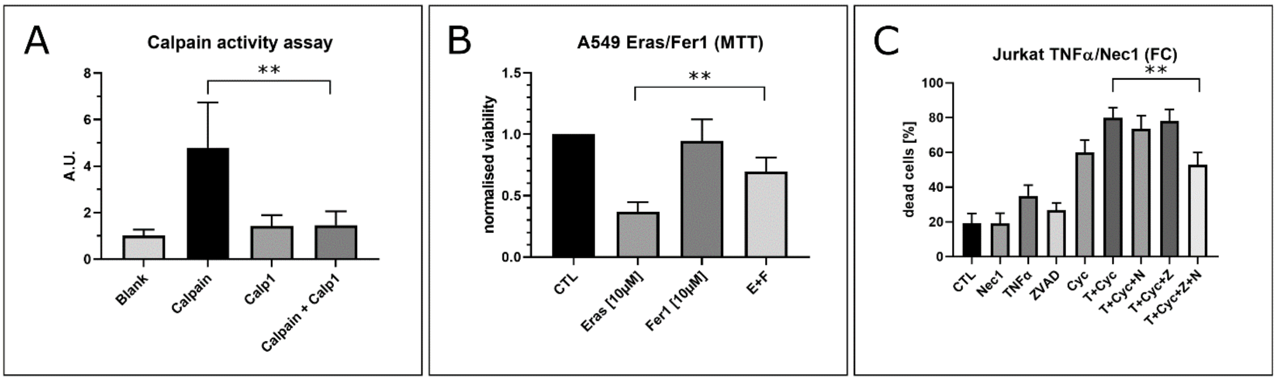

Figure S1. Testing efficacy of cell death inhibitors. (A) Ac-Lys-Lys-Norleucinal (Calp1, 10 µM) was tested via fluorometric calpain activity assay as described. Calp1 inhibited calpain protease significantly (p=0.0037), F(3,12)=11.11; p=0.0009). (B) A549 cells were treated with erastin (ERAS, 10 µM), ferrostatin 1 (Fer1, 10 µM) or in combination (E+F) and compared to untreated cells. While Fer1 alone did not impact cell viability (p=0.864) as monitored via MTT assay, ERAS significantly reduced cell viability (p<0.0001), which was inhibited by addition of Fer1 (p=0.0014), F(3,16)=33.23; p<0.0001). (C) Jurkat cells were treated with necrostatin 1 (Nec 1, 10 µM), TNFα (T, 100 ng/ml) or ZVAD (10 µM) and combinations thereof together with cycloheximide (Cyc, 2 µg/ml) as previously described[51]. Cell death was assessed by flow cytometry (FC) as described. Presence of Nec1 (N) and ZVAD (Z) in T/Cyc-treated cells prevented necroptosis significantly (p=0.0012), F(7,16)=54.79, p<0.0001). Data are given as arithmetic mean ± SD of three experiments. Statistical signifance was calculated via one-way ANOVA and Tukey's test. ** .. p < 0.01.
