# Supplementary material for: Delineation of cell death mechanisms induced by synergistic effects of statins and erlotinib in non-small cell lung cancer cell (NSCLC) lines
Source: Sci Rep. 2020 Jan 22;10:959. doi: 10.1038/s41598-020-57707-2 (PMC6976657; doi:10.1038/s41598-020-57707-2)

Delineation of cell death mechanisms induced by synergistic effects of statins and erlotinib in non-small cell lung cancer cell (NSCLC) lines

Alexander Otahal[1], Duygu Aydemir[1,2], Erwin Tomasich[1], Christoph Minichsdorfer[1]

[1] Anna Spiegel Research Facility, Internal Medicine I, Oncology Division, Medical University of Vienna, Lazarettgasse 14, 1090 Vienna

[2] Department of Medical Biochemistry, School of Medicine, Koc University, Istanbul, Turkey, Koc University Research Center for Translational Research (KUTTAM), Istanbul, Turkey; before 2016: Anna Spiegel Research Facility, Internal Medicine I, Oncology Division, Medical University of Vienna, Lazarettgasse 14, 1090 Vienna

Contributing author, alexander.otahal@meduniwien.ac.at (ORCID: 0000-0003-3705-0039)

Contributing author, daydemir16@ku.edu.tr (ORCID: 0000-0002-6449-2708)

Contributing author, erwin.tomasich@meduniwien.ac.at

Corresponding author, christoph.minichsdorfer@meduniwien.ac.at

(ORCID: 0000-0002-5455-9139)

Figure 1 – Supplementary information

Loading scheme for Calu6 and A549 blots:

Lane 1	Lane 2	Lane 3	Lane 4	Lane 5	Lane 6	Lane 7
Marker	Untreated	P100	P100+Mev	Mev	F100	F100+Mev

Loading scheme for H1993 blot:

Lane 1	Lane 2	Lane 3	Lane 4	Lane 5	Lane 6	Lane 7
Untreated	P100	P100+Mev	Mev	F100	F100+Mev	Marker

Calu6 PARP (upper bands), beta-actin (lower bands)

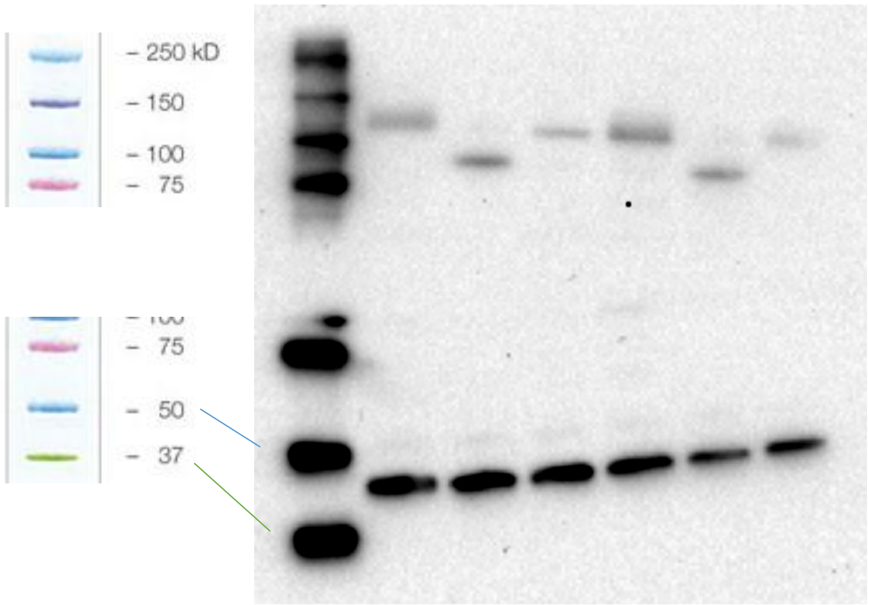

H1993 PARP (upper bands), beta-actin (lower bands)

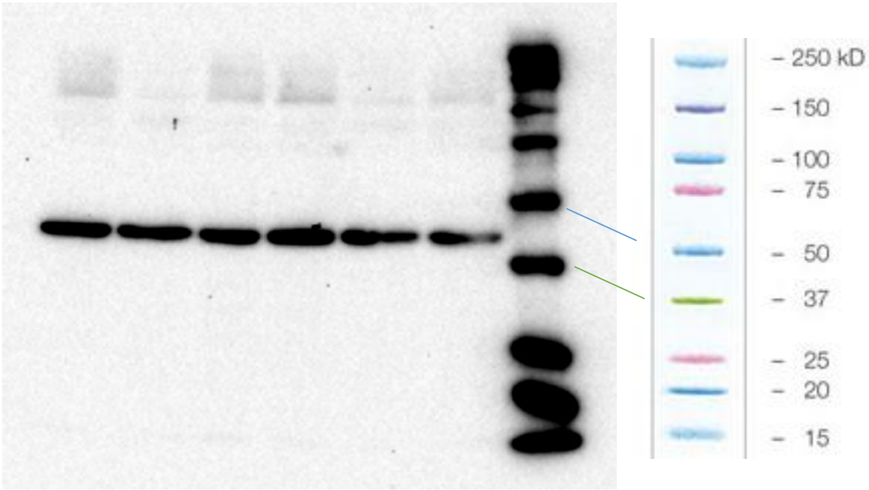

A549 PARP (upper bands), beta-actin (lower bands)

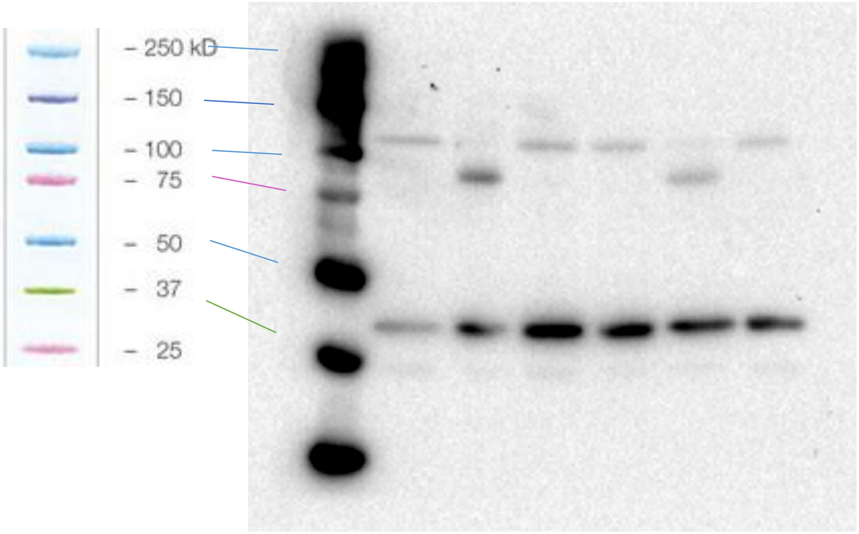

Figure 4 - Supplementary information

Loading scheme for all blots from figure 4:

Lane 1	Lane 2	Lane 3	Lane 4	Lane 5	Lane 6	Lane 7
Marker	Untreated	E5	F10	P10	E5F10	E5P10

Calu6 phospho-AKT

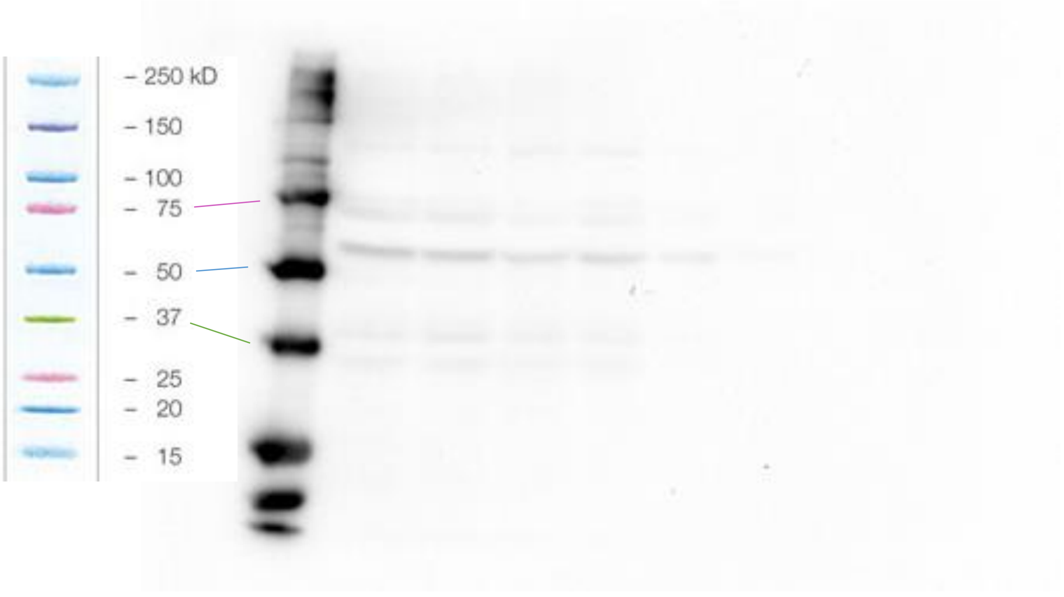

Calu6 AKT

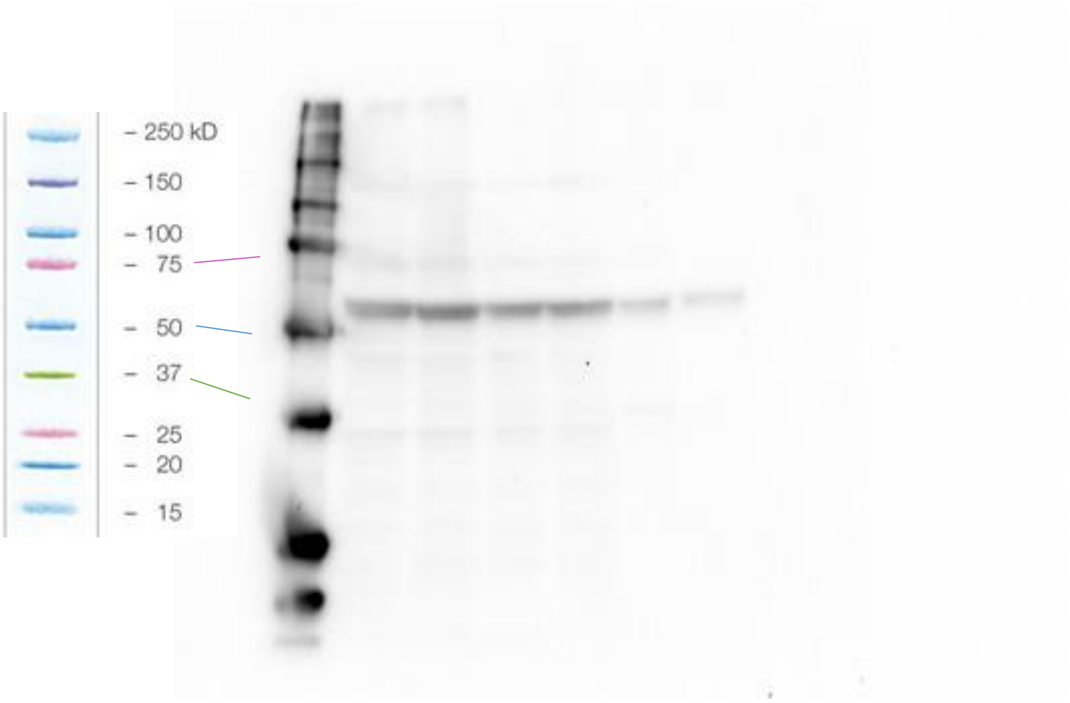

Calu6 phospho-ERK

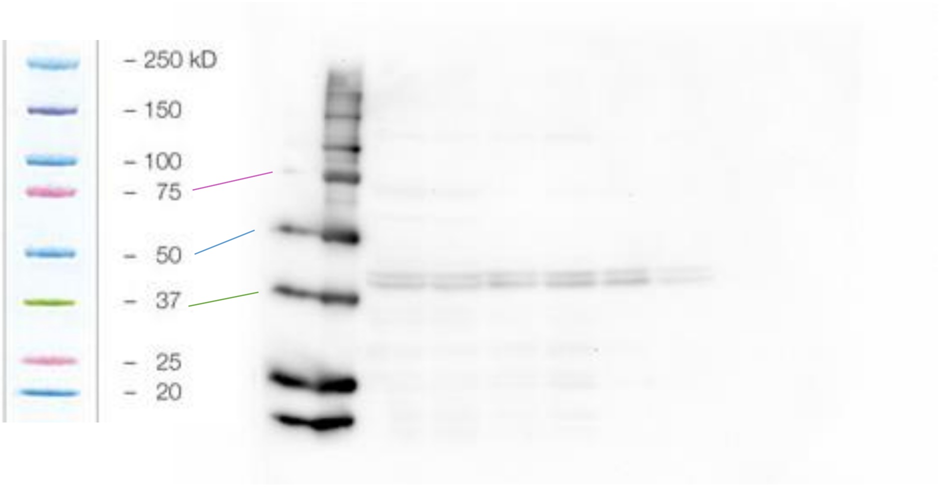

Calu6 ERK

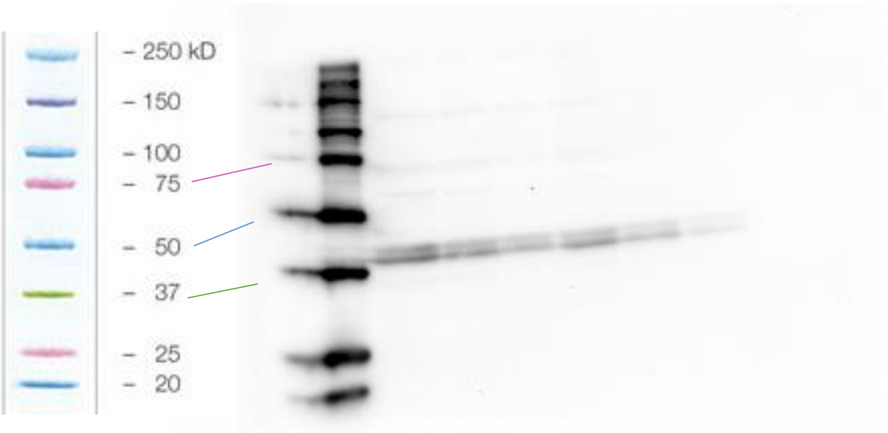

Calu6 beta-actin

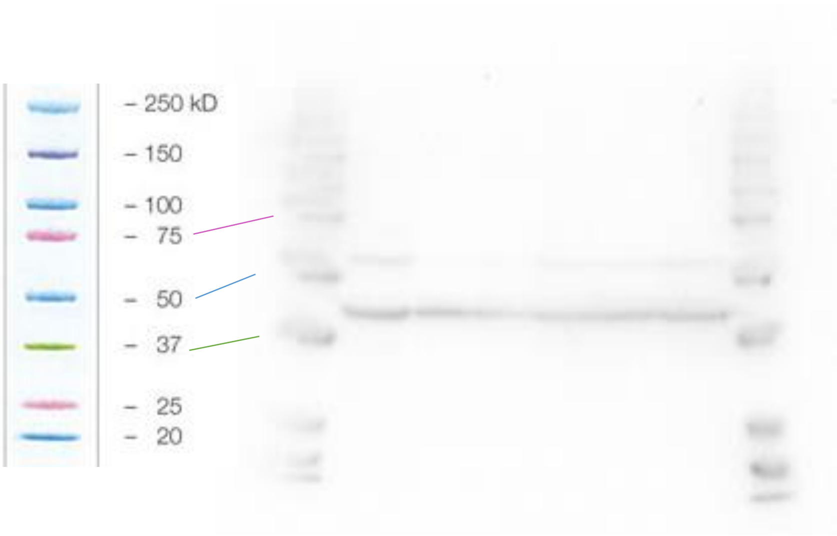

H1993 pospho-AKT

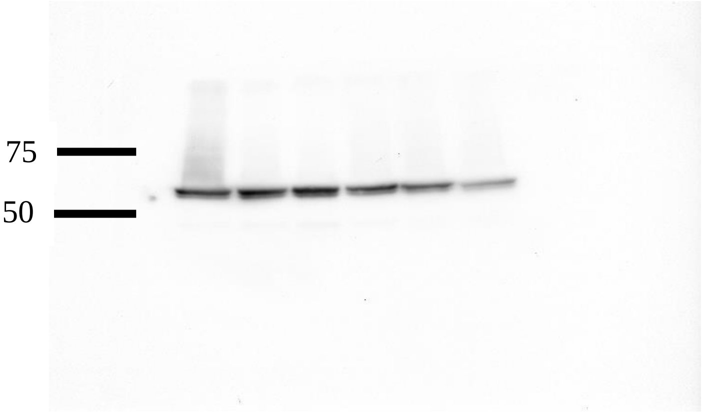

H1993 AKT

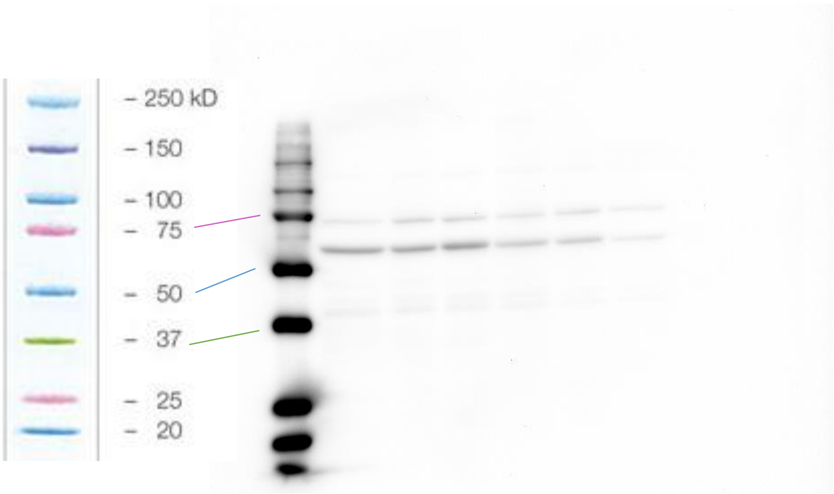

H1993 phospho-ERK

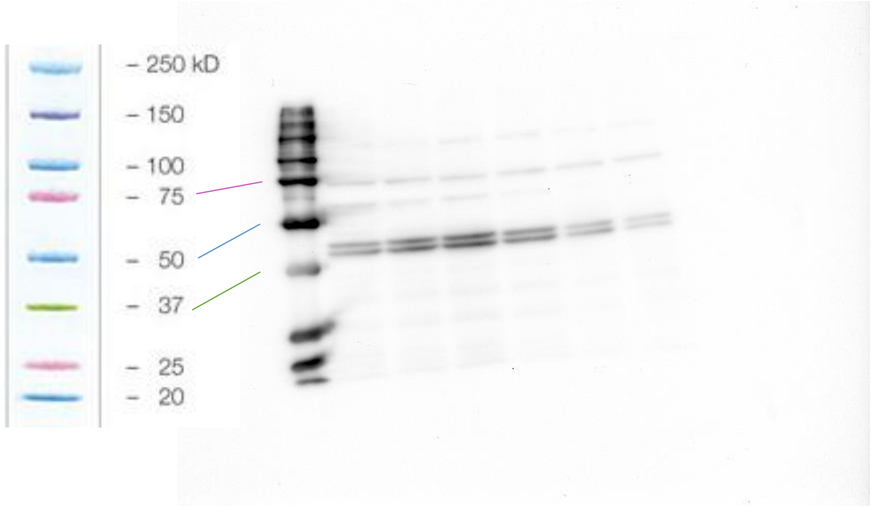

H1993 ERK

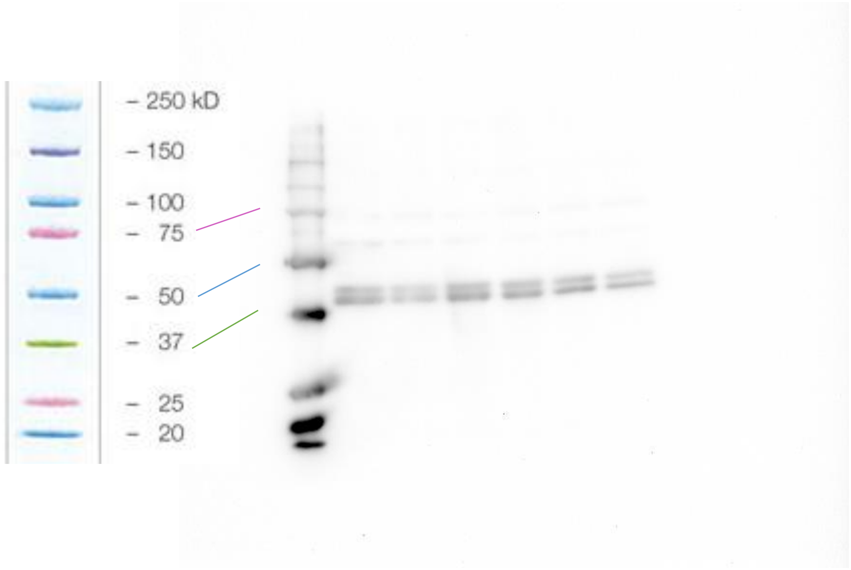

H1993 beta-actin

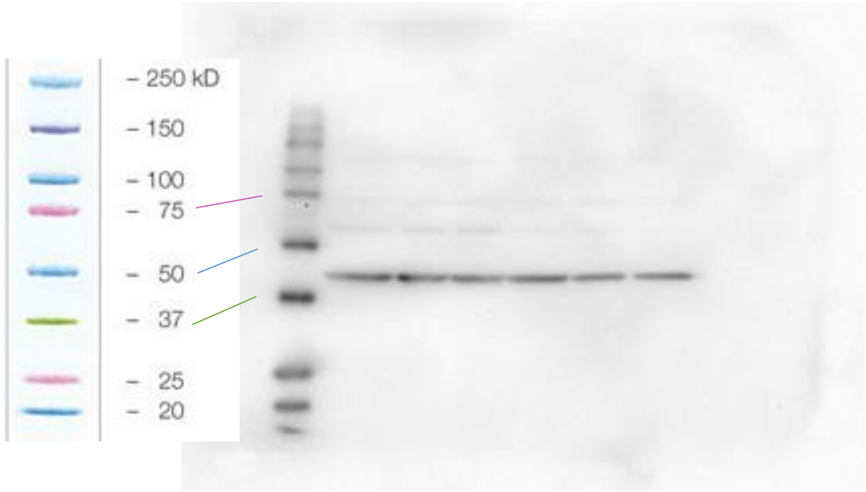

A549 phospho AKT

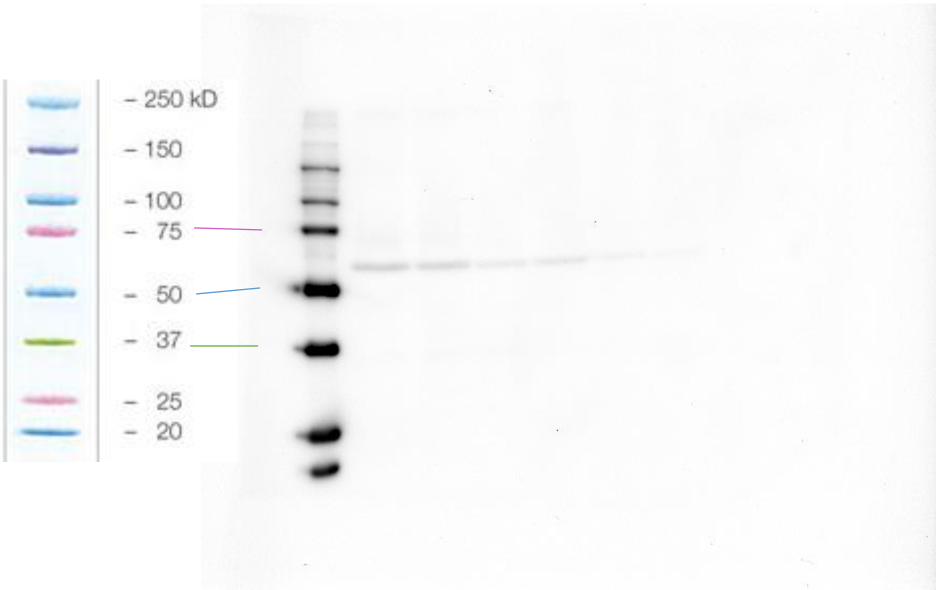

A549 AKT (upper bands)

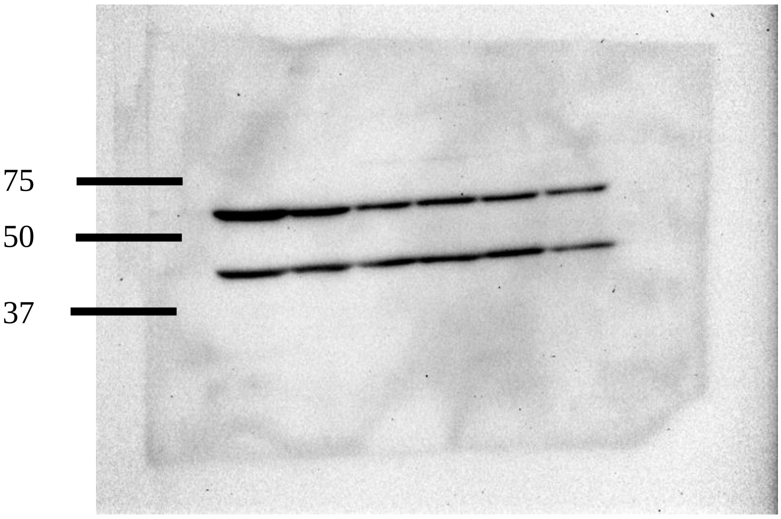

A549 phospho ERK

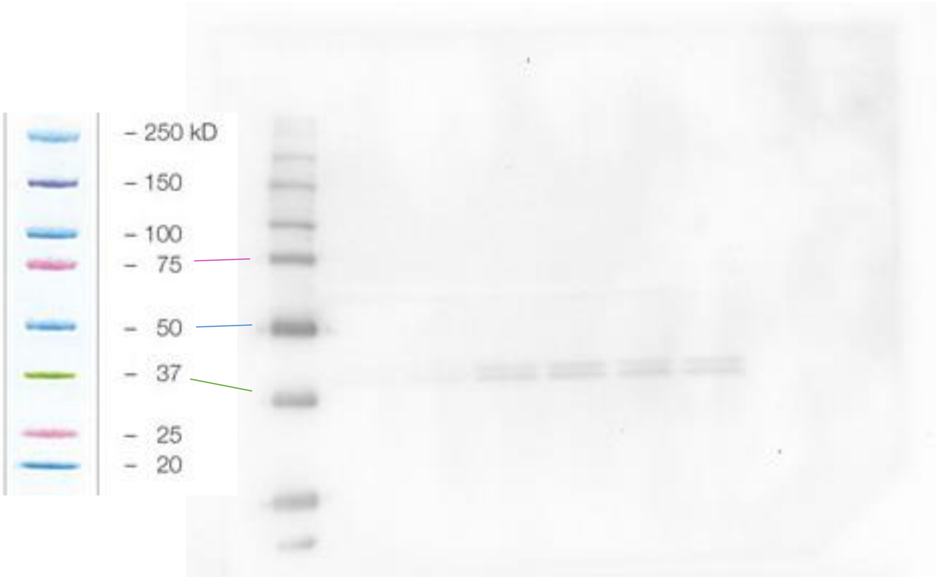

A549 ERK

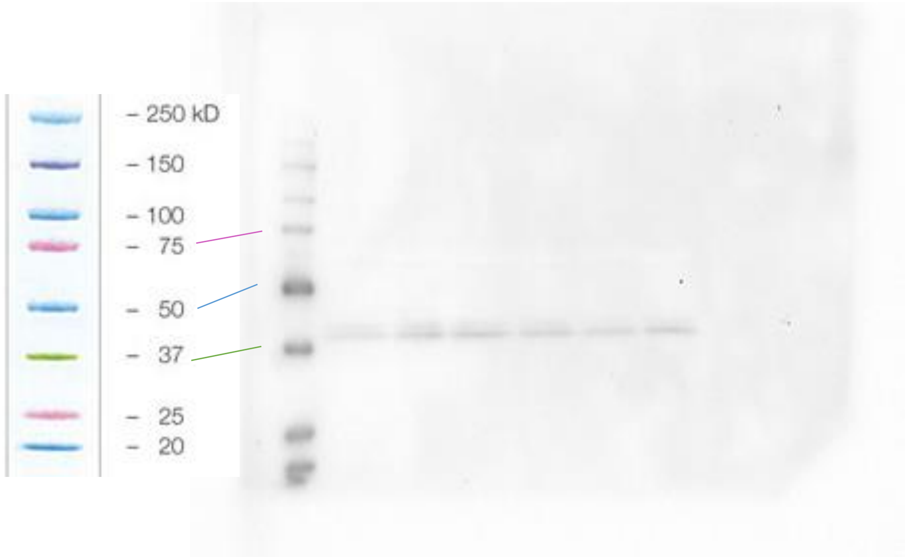

A549 beta actin (lower bands)

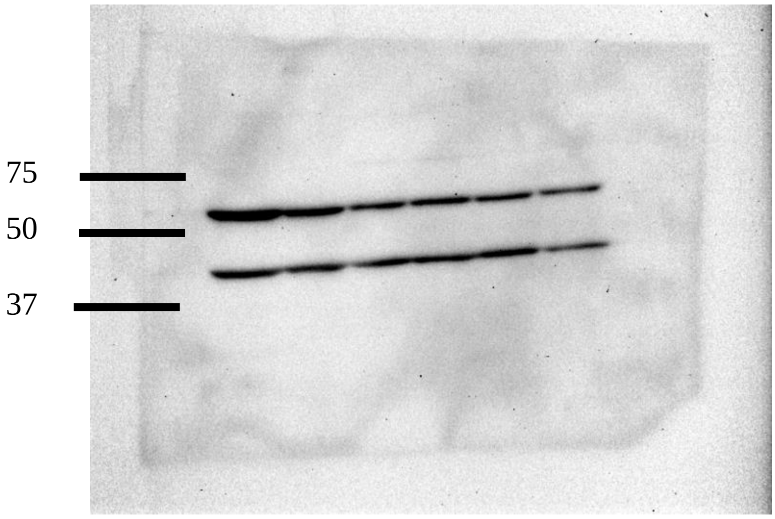

Supplement: Supplementary file 2 — Western Blots. [file 41598_2020_57707_MOESM2_ESM.pdf]
